# Patient Understanding of Uterine Fibroids and the Different Surgical Approaches to Hysterectomy

**DOI:** 10.1089/whr.2020.0040

**Published:** 2020-08-17

**Authors:** Anastasia Belilovets, Nithya Gopal, Erica Stockwell, Jasmine Pedroso, Joy Brotherton, K. Warren Volker, David Howard

**Affiliations:** ^1^ Obstetrics and Gynecology, Sunrise Health Graduate Medical Education Consortium, Las Vegas, Nevada, USA.; ^2^ Las Vegas Minimally Invasive Surgery/Women's Pelvic Health Center (A Davita Medical Group), Las Vegas, Nevada, USA.; ^3^ Obstetrics and Gynecology, University of Nevada Las Vegas, Las Vegas, Nevada, USA.; ^4^ Obstetrics and Gynecology, Harbor-UCLA Medical Center, Torrance, California, USA.; ^5^ Obstetrics and Gynecology, David Geffen School of Medicine, University of California Los Angeles, Los Angeles, California, USA.

**Keywords:** health literacy, hysterectomy, uterine fibroids

## Abstract

***Objective:*** The purpose of this study was to assess understanding of the hysterectomy procedure and uterine fibroids among women in a general gynecology clinic.

***Materials and Methods:*** This was an anonymous cross-sectional survey. We adapted and pilot tested a survey instrument designed to assess understanding of the hysterectomy procedure and of uterine fibroids. The final version of the survey consisted of basic demographic questions, followed by 28 knowledge questions (Canadian Task Force Classification II-2). The survey was disseminated to women in the waiting room of one of our gynecology clinics. The patient population included women 18 years and older.

***Results:*** The mean age of respondents was 33.5 years old. In total, 69.5% of the respondents had at least some college education. In the group of questions related to different types of hysterectomies, the most poorly answered question was “Which type of hysterectomy has the highest risk of damage to the bladder?” Less than 40% of the respondents were able to identify a laparoscopic and robotic hysterectomy based on a written description. Of questions about uterine fibroids, the most poorly answered question was whether cancer that looks like fibroids is common, with >90% of the respondents incorrectly thinking that cancer that resembled fibroids is common. More than half of respondents did not know what a fibroid is.

***Conclusions:*** In this analysis of the understanding of the hysterectomy procedure and fibroids among an educated population, overall understanding was poor. Specific areas where knowledge was particularly poor were the different ways of doing a hysterectomy and uterine fibroids.

## Introduction

Over 20 million women have had a hysterectomy, making hysterectomies the most common gynecological procedure, with >600,000 performed each year.^1,2^ Leiomyoma, abnormal uterine bleeding, benign ovarian neoplasm, endometriosis, pelvic organ prolapse, and gynecologic cancer are the main reasons women get hysterectomies.^3^ There are multiple surgical approaches to hysterectomy, including abdominal, vaginal, laparoscopic, and robotic.

Leiomyomas are the most common pelvic tumors in women, resulting in a great impact on quality of life, morbidity, and economic burden. Risk factors for leiomyomas include early menarche, nulliparity, family history, and obesity.^4^ Alternatives to hysterectomy exist for most indications and, therefore, patient understanding of the procedure is crucial for informed decision making. Some alternatives to hysterectomy for leiomyomas include medication, uterine artery embolization, and myomectomy.^5,6^

Compared with abdominal hysterectomy, minimally invasive hysterectomies are associated with decreased postoperative intravenous analgesia requirements, quicker return to daily activities, and shorter hospital stays.^6^ Overall hospital cost is about the same, due to longer operating times but shorter hospital stays with minimally invasive surgery.^6,7^ Multiple factors, such as uterine size, accessibility, mobility, adhesions, uterine shape, uterine pathology, and surgeon experience determine the optimal route of surgery.^8^

A patient's understanding of the hysterectomy procedure, including the different surgical approaches, is critical for decision making and adequate preoperative counseling. To our knowledge, only one prior study has specifically assessed patient understanding of the different approaches to gynecologic surgery.^9^ However, that study did not assess women's understanding of fibroids. The primary objective of our study was to assess, at the population level, women's general understanding of the hysterectomy procedure and of uterine fibroids.

## Materials and Methods

### Study background

The authors of this study have great interest in improving health literacy among women in the population at large. With hysterectomy being the most common gynecological surgical procedure, this was the area of focus for the study. We were concerned by the lack of a comprehensive educational tool, such as a video or visual aid that focused on either the hysterectomy procedure or uterine fibroids. We have since developed a tool, in the form of a video that is aimed at educating women about the hysterectomy procedure and uterine fibroids. To prove that our video improves knowledge, we developed an assessment tool/questionnaire to accompany the video.

### Survey development

We started survey development by searching for any pre-existing validated instrument that assessed women's understanding of the hysterectomy procedure. We found one that was developed by Dr. Karen Finck.^10^ Dr. Finck's instrument was developed >20 years ago when laparoscopic and robotic hysterectomies were not surgical options. She developed her instrument as part of her master's thesis, but the thesis was not published. We contacted Dr. Finck, and she provided written permission to use and adapt her survey for our study.

We adopted Dr. Finck's instrument to reflect newer surgical approaches, such as laparoscopy and robotic assisted laparoscopy. The next step was to conduct a focus group, led by an expert sociologist, to pilot test both the new video we created and the adapted survey instrument. The objective of evaluating the video and the tool in the same focus group was to ensure that we would end up with an assessment tool that reflected the content covered in the video and vice versa. If the assessment tool asked questions not covered in the video, then it would not be an appropriate way to assess the effect of the video on knowledge and understanding.

The focus group began with the participants completing our adapted questionnaire, then watching the video. The sociologist led a discussion with the participants about the video, focusing on what they did not know before watching the video, what they learned from watching the video, and what they still did not understand after watching the video. Following the focus group, the sociologist provided detailed recommendations for improving the instrument and the video to ensure that the video addressed any newly discovered knowledge gaps and that the assessment tool accurately reflected the content of the video. We incorporated all the sociologist's recommendations and refined the video and the assessment.

### Study design and population

This was an anonymous cross-sectional survey administered in paper form to patients presenting to a general obstetrics and gynecology clinic. This clinic is part of a large privately owned multispecialty practice. This survey is structured as a population-based survey. The goal was for respondents to represent a broad array of women, so we did not restrict the survey to women presenting for preoperative counseling for hysterectomy or consultation for abnormal bleeding. In summary, our study population was an unselected group of nonpregnant adult women presenting for any reason to a large gynecology practice. We did not exclude women based on the reasons they presented for care.

### Survey content

Dr. Finck's original survey is shown in Supplementary Appendix S1. Our survey, created after adapting Dr. Finck's survey and subjecting it to focus group testing and further refinements, is shown in Supplementary Appendix S2. The refined version of the video we created can be viewed in Supplementary Video S1. The final version of our survey/assessment tool consisted of basic demographic questions, followed by 28 knowledge questions. The survey began with 10 demographic questions focusing on age, ethnicity, educational background, gynecological history, and surgical history. To our knowledge, all survey participants were proficient in reading and speaking English. The first several knowledge questions focused on female anatomy, physiology, and knowledge of fibroids. Then, the next several questions asked about types of hysterectomy (abdominal, vaginal, laparoscopic, and robotically assisted), whereas the next set of questions focused on what happens after a hysterectomy, indications for a hysterectomy, complications of the procedure, and the actual hysterectomy procedure itself. The survey concluded with questions about the risks of undergoing a hysterectomy. All questions were in multiple choice format and included the option of “I don't know” for most questions. Participants were asked to select one answer for all questions. If participants selected “I don't know” as their response to a question, it was scored as incorrect.

### Survey dissemination

To minimize bias, we intentionally designed this initial study to consist of anonymous survey responses. We did not want any participant to be identifiable. Each survey had a number written on the top right corner of every page to distinguish each participant's response from the next during data entry. The paper surveys were placed inside an envelope with a pencil. Attached to the survey was an information sheet approved by the institutional review board that informed the patient about the study, the principal investigator, and the anonymity of the survey. This information sheet also clearly stated that, if the participant did not want to consent to being in the study, they should refrain from completing the survey and hand back the envelope. We did not compile information on who declined to participate in the survey. We obtained a waiver of signed consent from the institutional review board.

The envelopes were disseminated by medical assistants so that participants could complete the survey while sitting in the examination room and waiting for the staff gynecologist. Depending on the length of time from entry to the room and when the staff gynecologist arrived, patients either returned the envelopes before the visit started or after the visit ended.

### Exclusions

Women under the age of 18 years were excluded.

### Ethics

We obtained approval from the Touro University of Nevada Institutional Review Board to conduct this study. Because we wanted to conduct the survey anonymously, we applied for and received a waiver for signed consent. The information sheet attached to the questionnaire was reviewed and approved by the IRB. IRB approval: IRB12-14-16C November 21, 2016.

### Survey validation

The face validity of the developed survey was determined by the expert research team who conducted the study. The content validity was determined by the consensus of the research team and the feedback from the focus group. The reliability of the survey was evaluated by calculating the Cronbach alpha coefficient.

### Statistical analysis

All analyses were conducted with the use of STATA (version 15; STATA Corp., College Stations, TX). Our primary outcome, the total percentage of questions answered correctly, was a continuous variable. To assess the association between the outcome and various demographic factors, we used the analysis of variance (ANOVA) test. Survey questions fell into four discrete domains: types of hysterectomies, uterine fibroids, risks of hysterectomy, and life after hysterectomy. In addition to summarizing performance on each individual question in each domain, we also calculated the raw domain score for each participant and calculated the mean for all participants for each domain. To compare the four mean domain scores, we also used the ANOVA test. Statistical significance was defined as a *p*-value of <0.05.

### Sample size determination

Our primary aim was to assess general knowledge and to obtain enough survey responses to perform analyses, specifically the calculation of Cronbach's alpha, to further validate our knowledge instrument. Our primary aim was not to compare two groups of patients in terms of knowledge, so there was no predetermined “effect size” for us to use for determining sample size. Instead of the traditional “power calculation,” we set a predetermined target of at least 200 survey responses.

## Results

We collected 200 survey responses. As measured by the Flesch–Kincaid reading scale, the overall grade level of our survey questions was 8.1. The mean respondent age was 33.5 years old (95% confidence interval 31.6–35.4). Of the 200 respondents, 43.5% were Caucasian, 31.0% were Hispanic, 11.0% were Asian/Pacific Islander, and 10.5% were African American. In terms of education, 69.5% of the respondents had at least some college education. In terms of gynecological history, 9.0% had completed menopause, 6.0% had a prior hysterectomy, and 18.5% had a prior laparoscopic surgery (Table 1).

**Table 1.** Demographic Characteristics of Survey Respondents

Age, mean [95% CI]	33.5 [31.6–35.4]
Ethnicity, *n* (%)	
African American	21 (10.5)
Asian/Pacific Islander	22 (11.0)
Caucasian	87 (43.5)
Hispanic	62 (31.0)
Other	7 (3.5)
Education, *n* (%)	
Less than high school	12 (6.0)
High school	45 (22.5)
Some college	57 (28.5)
Master's or higher	17 (8.5)
Completed menopause	18 (9.0)
Had prior hysterectomy	12 (6.0)
Had prior laparoscopic surgery	37 (18.5)
Had prior cesarean section	40 (20)
Had prior vaginal delivery	69 (34.5)

In Table 2, we illustrate the association between demographic characteristics and performance on the survey. We found that there was an association between age and performance on the test. Specifically, women over the age of 30 years had a mean score of 16.6–16.8 out of 28 (59.3%–60% correct), whereas women <21 years old only scored an average of 13.5 out of 28 (48.2% correct; *p* = 0.03). In terms of ethnicity, Caucasians and African Americans scored the highest, with a mean score of 16.7 (59.6% correct) and 17.0 (60.7% correct), respectively, whereas Hispanics only scored an average of 13.9 out of 28 (49.6% correct; *p* = 0.03). Women with a history of any surgery scored significantly higher than women with no history of surgery (*p* = 0.001). Similarly, those with a prior laparoscopic surgery scored higher than those who never had a laparoscopic surgery (*p* = 0.02). Of note, there was no association between education level and mean test score.

**Table 2.** Performance on Hysterectomy Knowledge Test by Demographic Characteristics

	Score on the hysterectomy knowledge test (Maximum score possible is 28)	p^a^
Age (years)		0.03
Less than 21	13.5 (11.3–15.6)	
21–30	14.8 (13.4–16.1)	
31–50	16.6 (15.3–17.9)	
51 or older	16.8 (15.1–18.6)	
Ethnicity		0.03
African American	17.0 (15.2–18.8)	
Asian/Pacific Islander	15.4 (13.5–17.2)	
Caucasian	16.7 (15.4–17.9)	
Hispanic	13.9 (12.5–15.4)	
Other	13.7 (9.1–18.3)	
Education		0.15
Less than high school	12.4 (9.2–15.7)	
High school	15.0 (13.5–16.6)	
Some college	15.4 (14.0–16.8)	
College graduate	16.6 (15.3–18.0)	
Master's or higher	16.5 (13.4–19.7)	
Completed menopause		0.15
Yes	17.4 (14.9–19.9)	
No	15.4 (14.6–16.2)	
Had prior hysterectomy		0.13
Yes	17.9 (14.8–21.0)	
No	15.4 (14.6–16.2)	
Had prior laparoscopic surgery		0.02
Yes	17.6 (15.8–19.5)	
No	15.3 (14.4–16.2)	
Had prior cesarean section		0.20
Yes	16.6 (14.9–18.3)	
No	15.3 (14.4–16.2)	
Had prior vaginal delivery		0.86
Yes	15.5 (14.1–16.9)	
No	15.6 (14.7–16.6)	

^a^ All *p*-values obtained from one-way ANOVA tests.

In Table 3, we show the performance of the study population on each domain of the test and the reading level of each question. In our judgment, 24 out of the 28 questions fell into 4 domains. In the group of questions related to the different types of hysterectomies, the most poorly answered question was the question on which type of hysterectomy had the highest risk of damage to the bladder—only 8.0% answered this question correctly. The reading level of this question was lower than an eighth-grade level. Also of note, within this domain, <40% of the patients were able to identify a laparoscopic and robotic hysterectomy based on a written description.

**Table 3.** Performance of the Study Population on Each Hysterectomy Knowledge Question Within Four Main Domains

Domain	Questions	Flesch–Kincaid reading level	Percent who got it correct
Different types of hysterectomies	In this type of hysterectomy, the uterus is removed through a large cut on the abdomen (belly).	9.7	58.5
	In this type of hysterectomy, the uterus is removed through the vagina with no cuts in the abdomen (belly).	11.0	52.5
	In this type of hysterectomy, the surgeon controls a machine to do the surgery through tiny cuts on the abdomen (belly). The uterus is then removed through either the abdomen or the vagina.	10.5	38.0
	In this type of hysterectomy, the surgeon works directly through small cuts made on the abdomen (belly) and the uterus is either removed through the abdomen or through the vagina.	15.3	39.0
	Which type of hysterectomy has the longest recovery time?	10.2	53.0
	Which type of hysterectomy has the highest risk of damage to the bladder?	7.6	8.0
Uterine fibroids	Fibroids are growths on or in the uterus that can cause it to grow bigger.	5.2	48.0
	Fibroids are a type of cancer.	2.4	58.5
	There is a type of cancer that can look like a fibroid.	2.8	40.0
	Cancer that looks like fibroids is common.	3.9	8.5
	Fibroids are not a common reason for women to have a hysterectomy.	8.7	43.5
Risks of hysterectomy	State whether the following are risks of undergoing a hysterectomy surgery.	12.3	
	Bleeding		79.5
	Blood clots in the legs		62.0
	Blood clots in the lungs		53.0
	Infection		79.0
	Damage to other organs (bladder, bowel)		62.0
	Hair loss		48.0
	Injury to nerves		52.5
	Death		55.5
Life after hysterectomy	After a hysterectomy, a woman's menstrual period…	14.1	66.0
	After having a hysterectomy, a woman will still be able to get pregnant.	9.4	87.5
	When you have a hysterectomy, the ovaries are always removed.	9.5	43.0
	After a hysterectomy, a woman cannot have sex ever again.	10.7	91.0

With respect to the domain of questions on uterine fibroids, the most poorly answered question was the question that asked whether cancer that looks like fibroids is common. Over 90% of the respondents incorrectly thought that cancer that resembled fibroids is common, and more than half the respondents did not know what a fibroid was.

In terms of the domain of questions related to the risks of hysterectomy, the most poorly answered question was the question related to whether hair loss is a risk of undergoing hysterectomy. Over half of the respondents incorrectly thought that hair loss was a risk of undergoing hysterectomy.

With respect to the domain of questions related to life after hysterectomy, the most poorly answered question asked whether the ovaries are always removed during a hysterectomy—only 43% of the respondents answered correctly.

When we scored all the respondents by how they performed on each domain, the domains with the best performance were the domains related to risk of hysterectomy and life after hysterectomy, with respondents getting at least 60% of the questions correct in these two domains. Participants scored highest on these two domains, even though the reading levels of the questions in these two domains were above an eighth-grade level (Table 3).

In contrast, performance on the domains regarding the types of hysterectomies and uterine fibroids was much poorer, with the average respondent only getting just >40% of the questions correct in each domain. The poor performance on the domain of questions related to uterine fibroids is notable, considering the reading level of these questions was the lowest of all the domains (Table 3).

We conducted the ANOVA test to determine whether the mean scores on each of the four domains were equal. The results of the ANOVA test revealed that the difference between performance on the four domains was statistically significant (*p* < 0.001). Finally, the overall Cronbach alpha coefficient for the 28-item knowledge assessment portion of our survey was 0.85.

## Discussion

This pilot study, to the best of our knowledge, represents the first attempt to create a validated assessment tool to evaluate women's understanding of both the hysterectomy procedure and uterine fibroids. Our primary finding was that, among patients presenting to a gynecology clinic in a large diverse city, there was a poor overall level of understanding of the hysterectomy procedure. Understanding of the different approaches to hysterectomy and understanding of uterine fibroids was particularly poor among the patient participants. We consider this study as more hypothesis generating, rather than definitive. We hope that other researchers will replicate our study to validate our findings. In terms of reliability, the overall Cronbach's alpha of 0.85 suggests that, although our assessment tool could be improved, it had an acceptable level of reliability.

Today, with so many technological advances, we believe health literacy becomes even more critical to informed consent. Do patients really understand the differences between a vaginal, abdominal, laparoscopic, and robotic hysterectomy? Our study suggests they do not. Furthermore, despite uterine fibroids being one of the leading indications for hysterectomy, understanding of uterine fibroids was poor among the participants in this survey. It was surprising that understanding of the hysterectomy procedure among patient participants was so poor, even though ∼70% had at least some college education.

The results of two prior studies^11,12^ are broadly consistent with our study. However, both prior studies only addressed women's knowledge of general aspects of hysterectomy and female anatomy. Neither addressed women's understanding of the different surgical approaches to hysterectomy, the differences in specific risks between each hysterectomy approach, or women's understanding of uterine fibroids. A recent randomized trial exposed women preparing for a hysterectomy to standard face-to-face preoperative counseling versus a video presentation, followed by standard preoperative face-to-face counseling.^13^ The study found that women in the control arm were less likely to receive counseling about the risks of hysterectomy, such as thrombosis, and were less likely to receive counseling about postoperative expectations and the need for possible hormone replacement therapy. The study also found that the improvement in women's comprehension of the hysterectomy procedure was greater after exposure to a video presentation versus after standard counseling. This finding supports our *a priori* belief that the use of visual aids can augment and strengthen preoperative counseling and improve informed consent.

The main limitation of our study is that, although the patient population was diverse, the patients all came from a single clinic. Our patient population appears to be very educated, with ∼70% completing at least some college. This would be a limitation in our study if the women scored highly. However, understanding of hysterectomy was still poor among this patient population. Some of our knowledge questions were at a higher than eight-grade reading level, but the domain of questions with the worst performance had the lowest reading level (lower than fifth grade). We do not believe that education influenced the response to higher level question. The two domains with the best performance were at a higher than 10th-grade level. The poor performance on the domain of questions related to uterine fibroids, despite the low reading level of the questions and the high educational level of our population, is concerning. This suggests to us that there may be a real deficit in the understanding of uterine fibroids. Another limitation is that we had a small proportion of women who had a prior hysterectomy. Question 7 was the only question with “not applicable” as a response, this could be a factor in why so many women got that question wrong. This is a poorly worded question, and since this is a pilot study, in the future, we would omit the “not applicable” option.

## Conclusions

We created an assessment tool to evaluate women's understanding of the hysterectomy procedure and uterine fibroids that has promising characteristics, such as a Cronbach alpha coefficient of 0.85 that suggests high reliability. In this analysis of understanding of the hysterectomy procedure and fibroids, we found that overall understanding was poor. The specific areas where knowledge was poorest were the different ways of doing a hysterectomy and knowledge of uterine fibroids. We are cautious in the generalizability of our conclusions because of the limitations already stated. Further work needs to be done to stringently validate the comprehensive assessment tool we developed and used in this study so that the effectiveness of the educational video we created can then be assessed. In addition, understanding of the hysterectomy procedure and uterine fibroids needs to be assessed among non-English–speaking women as well. This will require translation of both the assessment tool and educational tool into other languages, followed by validation and further research studies.

## Supplementary Material

Supplemental data

Supplemental data

Supplemental data

## Abbreviation Used

ANOVA: analysis of variance
