# Supplementary material for: Patient Understanding of Uterine Fibroids and the Different Surgical Approaches to Hysterectomy
Source: Womens Health Rep (New Rochelle). 2020 Aug 17;1(1):252–8. doi: 10.1089/whr.2020.0040 (PMC7784805; doi:10.1089/whr.2020.0040)
Supplement: Supplemental data [file Supp_App1.pdf]

APPENDIX G HYSTERECTOMY KNOWLEDGE INTERVIEW SCHEDULE

HYSTERECTOMY QUESTIONS

HYSTERECTOMY IS THE REMOVAL OF A WOMAN'S UTERUS (OR WOMB). IN THIS QUESTIONNAIRE, HYSTERECTOMY DOES NOT INCLUDE THE REMOVAL OF A WOMAN'S OVARIES.

SECTION I

IN THIS SECTION, PLEASE CHECK THE BOX IN THE TRUE COLUMN IF YOU THINK THE STATEMENT IS TRUE. IF YOU THINK THE STATEMENT IS FALSE, THEN CHECK THE CORRESPONDING BOX IN THE FALSE COLUMN. IF YOU ARE UNCERTAIN ABOUT THE CORRECT ANSWER, PLEASE MAKE YOUR BEST GUESS.

EXAMPLES:

True	False	
(✓)	( )	0. Hysterectomy is the removal of a woman's uterus.
( )	(✓)	0. Hysterectomy includes the removal of a woman's ovaries.

NOW PLEASE ANSWER ALL THE QUESTIONS IN THIS SECTION.

True	False	
( )	( )	1. Most hysterectomies are performed because the woman has cancer.
( )	( )	2. Most hysterectomies are performed to save a woman's life.
( )	( )	3. Some hysterectomies are performed unnecessarily.
( )	( )	4. Hysterectomies will cure premenstrual tension (headaches and depression before a menstrual period).
( )	( )	5. Women get hysterectomies as a result of an over active sex life.
( )	( )	6. After hysterectomy, most women's desire for sex will end.
( )	( )	7. After hysterectomy, it is impossible for a woman to give birth to a child.
( )	( )	8. For many women, being able to have children is an important part of the way they see themselves.
( )	( )	9. If a woman has all the children she wants, a hysterectomy will have no emotional effect on her.
( )	( )	10. If a woman is unmarried and does not want children, a hysterectomy will have no emotional effect on her.
( )	( )	11. Women have the same emotional responses to having a hysterectomy as to having a tubal ligation (tubes tied).
( )	( )	12. After hysterectomy, many women to through a time of depression.
( )	( )	13. After a woman goes through menopause (change of life), a hysterectomy will not affect her emotionally.
( )	( )	14. After hysterectomy, most women must get psychiatric help.

A (G)

True	False	
( )	( )	15. After hysterectomy, women appear to age rapidly, getting grey hair and wrinkles.
( )	( )	16. After hysterectomy, women develope a more masculine appearance including a deeper voice and facial hair.
( )	( )	17. Many women gain weight after having a hysterectomy.
( )	( )	18. Many women get headaches for a period of time after a hysterectomy.
( )	( )	19. Many women get hot flashes for a period of time after hysterectomy.
( )	( )	20. Some women have urinary (bladder) problems temporarily after having a hysterectomy.
( )	( )	21. After hysterectomy, many women lose control of their bladder and bowels permenently.
( )	( )	22. Many women feel very tired for a period of time after a hysterectomy.
( )	( )	23. After a hysterectomy, a woman will never be as healthy as she was before.
( )	( )	24. After a hysterectomy, a woman will never be as physically strong as she was before.
( )	( )	25. A woman can usually return to work or household duties 6 to 8 weeks after she has had a hysterectomy.
( )	( )	26. Hysterectomies are as medically safe as tubal ligations (having your tubes tied).

SECTION II

IN THIS SECTION, PLEASE CHECK THE BOX BEFORE THE CORRECT ANSWER TO EACH QUESTION. CHECK ONLY ONE ANSWER FOR EACH QUESTION.

EXAMPLE:

0. A hysterectomy is the removal of a woman's
   - ( ) a. stomach.
   - (✓) b. uterus.
   - ( ) c. ovaries.

NOW PLEASE ANSWER ALL THE QUESTIONS IN THIS SECTION. CHECK ONLY ONE ANSWER FOR EACH QUESTION.

1. After a hysterectomy, a woman's menstrual period
   - ( ) a. stops entirely and never returns.
   - ( ) b. does not stop, but becomes irregular.
   - ( ) c. continues as it was before the hysterectomy.

A (G)

2. A woman's menopause (change of life)
   ( ) a. can be prevented by having a hysterectomy.
   ( ) b. will be brought on by a hysterectomy.
   ( ) c. will occur when it normally would.

3. After a woman reaches menopause (change of life)
   ( ) a. her uterus should be removed to prevent the woman from getting cancer.
   ( ) b. her uterus should remain in her body unless it becomes diseased.
   ( ) c. her uterus should never be removed.

4. After a hysterectomy, most women
   ( ) a. cannot have sexual intercourse ever again.
   ( ) b. can have sexual intercourse, but it will never feel the same to her.
   ( ) c. can have sexual intercourse and it will feel the same to her as it did before the hysterectomy.

5. After a hysterectomy, most women
   ( ) a. will not be able to have an orgasm again.
   ( ) b. will have an orgasm if she did before the surgery.
   ( ) c. will be able to have an orgasm even if she couldn't have one before the surgery.
   ( ) d. I am unsure what an orgasm is.

6. After a woman is recovered from a hysterectomy, her desire for sex will probably
   ( ) a. increase.
   ( ) b. decrease.
   ( ) c. remain the same as it was before the hysterectomy.

7. A man
   ( ) a. will not be able to have sexual intercourse with a woman who has had a hysterectomy.
   ( ) b. can have sexual intercourse with a woman who has had a hysterectomy but it will feel different to him.
   ( ) c. can have sexual intercourse with a woman who has had a hysterectomy and it will feel the same to him.

8. What is the general size of a non-pregnant uterus?
   ( ) a. an orange.
   ( ) b. a grapefruit.
   ( ) c. a large cantelope.

A (G)

9. When the uterus is removed from a woman's body

( ) a. there is an empty space where the uterus was.

( ) b. fluid fills in the space and the other organs move slightly to fill in the space.

( ) c. the doctor inserts a sponge-like substance in the empty space during surgery.

10. After a woman is recovered from hysterectomy, the ovaries

( ) a. no longer function.

( ) b. function at a reduced rate.

( ) c. function as they did before the hysterectomy.

11. Most hysterectomies are performed because

( ) a. the woman has fibroids (non-cancerous growths) in her uterus.

( ) b. the woman has cancer.

( ) c. most hysterectomies are done unnecessarily.

12. General recovery time for most major surgery is three months (until a person feels totally like themself again). What is the recovery time for a hysterectomy?

( ) a. 6 weeks.

( ) b. 3 months

( ) c. 1 year

( ) d. a woman never feels like herself again after a hysterectomy.

SECTION III

IN THIS SECTION PUT A CHECK IN THE BOX BEFORE ALL OF THE CORRECT ANSWERS TO EACH QUESTION. YOU MAY CHECK MORE THAN ONE ANSWER FOR EACH QUESTION.

1. What is the uterus responsible for in the body?

( ) a. it holds menstrual blood.

( ) b. it is where sexual intercourse occurs.

( ) c. it produces hormones.

( ) d. it is where a baby grows until birth.

( ) e. it controls a woman's sex drive.

2. What are the ovaries responsible for in the body?

( ) a. they hold menstrual blood.

( ) b. they produce hormones.

( ) c. they store and release eggs.

( ) d. they control a women's sex drive.

( ) e. they are where sexual intercourse occurs.
