# Supplementary material for: Patient Understanding of Uterine Fibroids and the Different Surgical Approaches to Hysterectomy
Source: Womens Health Rep (New Rochelle). 2020 Aug 17;1(1):252–8. doi: 10.1089/whr.2020.0040 (PMC7784805; doi:10.1089/whr.2020.0040)
Supplement: Supplemental data [file Supp_App2.pdf]

Supplementary Data

Supplementary Appendix S2

The following survey has been made to gather information on women's general knowledge of the hysterectomy procedure.

This survey was approved by the Institutional Review Board at Touro University.

The Principal Investigator is Dr. Jasmine Pedroso.

Please do not write your name or any other identifying information on any pages of the survey—it is meant to be confidential.

If you do not consent to doing this survey, then do not complete it. Return the envelope to the front desk.

Your confidential completion of the survey gives us consent to use your responses in our study.

———————————————————————

DEMOGRAPHICS: Please check or fill the appropriate box:

———————————————————————

1. What is your age:

____

2. What is your ethnicity:

☐ Caucasian/white
☐ Hispanic
☐ African American/black
☐ Native American
☐ Asian/Pacific Islander
☐ Other

3. What is the highest level of education you completed?

☐ Less than high school
☐ I started high school, but did not finish
☐ High school diploma or GED
☐ I started some college, but did not finish
☐ Completed college/bachelor degree
☐ Master's degree
☐ Doctorate

4. Are you planning on having a hysterectomy (removing the uterus/womb) within the next 6 months?

☐ Yes
☐ No
☐ I don't know

5. Have you completed menopause (no periods for at least 1 year)?

☐ Yes
☐ No
☐ I don't know

6. Have you had your uterus (womb) removed?

☐ Yes
☐ No
☐ I don't know

7. Have you ever had surgery?

☐ Yes
☐ No
☐ I don't know

8. Have you ever had laparoscopic surgery?

☐ Yes
☐ No
☐ I don't know

9. Have you ever had a cesarean section (C-section)?

☐ Yes
☐ No
☐ I don't know

10. Have you ever had a vaginal delivery?

☐ Yes
☐ No
☐ I don't know

———————————————————————

SECTION I: Please check one box for the most appropriate response.

EXAMPLES

The sky is blue.

☒ True
☐ False

———————————————————————

1. The uterus holds the baby during pregnancy.

☐ True
☐ False
☐ I don't know

2. The uterus is where bleeding comes from during your menstrual cycle (period).

☐ True
☐ False
☐ I don't know

3. Which body part makes female hormones?

☐ Uterus
☐ Liver
☐ Ovaries
☐ I don't know

4. Fibroids are growths on or in the uterus that can cause it to grow bigger.

☐ True
☐ False
☐ I don't know

5. Fibroids are a type of cancer.

☐ True
☐ False
☐ I don't know

6. There is a type of cancer that can look like a fibroid.

☐ True
☐ False
☐ I don't know

7. Cancer that looks like fibroids is common.
- ☐ True
- ☐ False
- ☐ I don't know
- ☐ Not applicable

Questions 8–11 are questions about different types of hysterectomies (ways of removing the uterus/womb).

8. In this type of hysterectomy, the uterus is removed through a large cut on the abdomen (belly).
- ☐ Abdominal
- ☐ Vaginal
- ☐ Laparoscopic
- ☐ Robotic
- ☐ Other
- ☐ I don't know

9. In this type of hysterectomy, the uterus is removed through the vagina with no cuts in the abdomen (belly).
- ☐ Abdominal
- ☐ Vaginal
- ☐ Laparoscopic
- ☐ Robotic
- ☐ Other
- ☐ I don't know

10. In this type of hysterectomy, the surgeon controls a machine to do the surgery through tiny cuts on the abdomen (belly). The uterus is then removed from either the abdomen or the vagina.
- ☐ Abdominal
- ☐ Vaginal
- ☐ Laparoscopic
- ☐ Robotic
- ☐ Other
- ☐ I don't know

11. In this type of hysterectomy, the surgeon works directly through small cuts made on the abdomen (belly) and the uterus is either removed from the abdomen or from the vagina.
- ☐ Abdominal
- ☐ Vaginal
- ☐ Laparoscopic
- ☐ Robotic
- ☐ Other
- ☐ I don't know

12. After a hysterectomy, a woman's menstrual period
- ☐ stops and never returns
- ☐ stops for a while and then returns
- ☐ does not stop, but becomes irregular
- ☐ continues as it was before the hysterectomy
- ☐ I don't know

13. Most hysterectomies are performed because a woman has cancer.
- ☐ True
- ☐ False
- ☐ I don't know

14. Fibroids are **not** a common reason for women to have a hysterectomy.
- ☐ True
- ☐ False
- ☐ I don't know

15. After having a hysterectomy, a woman will still be able to get pregnant.
- ☐ True
- ☐ False
- ☐ I don't know

16. When you have a hysterectomy, the ovaries are always removed.
- ☐ True
- ☐ False
- ☐ I don't know

17. After a hysterectomy, a woman cannot have sex ever again.
- ☐ True
- ☐ False
- ☐ I don't know

18. If the ovaries are left in place after the uterus is removed, they will no longer function.
- ☐ True
- ☐ False
- ☐ I don't know

State whether the following are risks of undergoing a hysterectomy surgery.

19. Bleeding ☐ Yes ☐ No
20. Blood clots in the legs ☐ Yes ☐ No
21. Blood clots in the lungs ☐ Yes ☐ No
22. Infection ☐ Yes ☐ No
23. Damage to other organs (bladder, bowel) ☐ Yes ☐ No
24. Hair loss ☐ Yes ☐ No
25. Injury to nerves ☐ Yes ☐ No
26. Death ☐ Yes ☐ No
27. Which type of hysterectomy has the **longest** recovery time?
- ☐ Abdominal
- ☐ Vaginal
- ☐ Laparoscopic
- ☐ Robotic
- ☐ I don't know

28. Which type of hysterectomy has the highest risk of damage to the bladder?
- ☐ Abdominal
- ☐ Vaginal
- ☐ Laparoscopic
- ☐ Robotic
- ☐ I don't know

Supplementary Video S1. Supplementary video aimed at providing information about the hysterectomy procedure and uterine fibroids.
